# Assessing animal affect: an automated and self-initiated judgement bias task based on natural investigative behaviour

**DOI:** 10.1038/s41598-018-30571-x

**Published:** 2018-08-17

**Authors:** Samantha Jones, Vikki Neville, Laura Higgs, Elizabeth S. Paul, Peter Dayan, Emma S. J. Robinson, Michael Mendl

**Affiliations:** 1 Centre for Behavioural Biology, Bristol Veterinary School, University of Bristol, Langford House, Langford, BS40 5DU UK; 2 Gatsby Computational Neuroscience Unit, University College London, 25 Howland Street, London, W1T 4JG UK; 3 School of Physiology, Pharmacology and Neuroscience, University of Bristol, Biomedical Sciences Building, University of Bristol, University Walk, Bristol, BS8 1TD UK; 4 Present Address: Department of Psychology, University of Portsmouth, King Henry Building, King Henry 1st Street, Portsmouth, PO1 2DY UK

## Abstract

Scientific methods for assessing animal affect, especially affective valence (positivity or negativity), allow us to evaluate animal welfare and the effectiveness of 3Rs Refinements designed to improve wellbeing. Judgement bias tasks measure valence; however, task-training may be lengthy and/or require significant time from researchers. Here we develop an automated and self-initiated judgement bias task for rats which capitalises on their natural investigative behaviour. Rats insert their noses into a food trough to start trials. They then hear a tone and learn either to stay for 2 s to receive a food reward or to withdraw promptly to avoid an air-puff. Which contingency applies is signalled by two different tones. Judgement bias is measured by responses to intermediate ambiguous tones. In two experiments we show that rats learn the task in fewer sessions than other automated variants, generalise responses across ambiguous tones as expected, self-initiate 4–5 trials/min, and can be tested repeatedly. Affect manipulations generate main effect trends in the predicted directions, although not localised to ambiguous tones, so further construct validation is required. We also find that tone-reinforcer pairings and reinforcement or non-reinforcement of ambiguous trials can affect responses to ambiguity. This translatable task should facilitate more widespread uptake of judgement bias testing.

## Introduction

Reduction and Replacement of animals used in science, and Refinement of experimental procedures to improve animal welfare (the 3Rs^1^), are important guiding principles in developing a humane approach to animal research (see: https://www.nc3rs.org.uk/). Improving animal wellbeing and minimising stress has also been proposed to enhance the scientific value of experimental studies^2–4^. However, in order to develop effective refinement strategies, accurate assessment of animal welfare is required so that welfare implications of existing practices and procedures, and of any refinements, can be evaluated. The assumption that non-human animals can subjectively experience negative affective (emotional) states, and hence suffer, underpins concerns for their wellbeing^2,5,6^. Affective states are conventionally defined in terms of two principal dimensions: arousal (how ‘activated’ the individual is), and valence (whether the individual is in a positive or negative state)^7,8^. From an animal welfare perspective, the valence dimension is critical.

Although we cannot directly measure the subjective component of affective states and do not know for certain which non-human species have such conscious experiences, we can use behavioural and physiological changes as proxy indicators of animal affect. One promising approach designed to assess affective valence involves testing an animal’s decision-making under ambiguity (‘judgement bias’)^9,10^. This is based on empirical investigations showing that people in negative affective states are more likely to make negative judgements about the meaning or outcome of ambiguous stimuli or situations^11,12^. From a theoretical perspective, background affective states (‘moods’) have been proposed to reflect cumulative experience of positive and negative events and hence act as Bayesian priors on the likelihood of such events occurring in the future. For example, experience of negative events predisposes a negative affective state and associated increased expectations of future negative events^8,13,14^. Priors of this sort are particularly influential when sensory evidence is weak and ambiguous.

A generic and translatable method for assessing judgement bias^9^ involves training subjects to make response P (e.g. pressing a lever) to a cue (e.g. tone of a specific frequency) predicting a positive outcome (e.g. food) in order to obtain that outcome, and response N (e.g. refraining from lever-pressing) to a different cue (e.g. a different frequency tone) in order to avoid a negative outcome (e.g. noise). Subjects are then presented with occasional ambiguous cues (e.g. tones of intermediate frequencies) with the prediction that animals in a more negative affective state are more likely to make response N – a negative judgement – than those in a more positive state. Although there are some null or apparently opposite findings in the more than 100 experimental studies in animal welfare science, neuroscience and psychopharmacology that have used variants of this generic method in mammals, birds and insects, the majority of published findings support the *a priori* prediction^10,15^.

A key barrier to widespread adoption of the approach is the often labour-intensive and time-consuming training of the basic discrimination task. Manual training of the task involves the researcher handling the animals between trials and/or operating apparatus to set up trials and deliver reinforcement as appropriate. Although it can be completed in a single 1–2 h session in dogs^16,17^, and in a small number of sessions in some other species^18–21^, it often takes considerably longer (e.g. over 20 sessions^22–24^; see^25^). Automation of training should decrease the time input required from researchers and minimise any effects of handling that may influence the animal’s affective state (e.g.^26,27^). Automated data collection should also eliminate any observer error or bias in recording animal decisions from direct or video observations, by allowing blinded data collection. It also allows many animals to be trained simultaneously, facilitating high-throughput experiments with large sample sizes. Successful automation would allow various methodological and theoretical issues related to the approach (e.g.^10,25,28^) to be tackled through series of experiments that can be completed quickly, and would increase the feasibility of using the method, and hence its wider uptake.

Automation is most readily implemented for laboratory animals where computer-controlled operant equipment is widely available. Several judgement bias studies of rats have used automated Skinner box or shuttle-box tasks but, whilst these free up researcher time, they have taken many sessions to train (e.g. 24–60^29–33^). Reasons for lengthy training times in automated operant tasks include the need for animals to learn non-naturalistic behavioural responses such as lever-pressing, and putative Pavlovian constraints on learning active and arbitrary responses (e.g. lever press) to avoid aversive stimuli^30^. Such constraints may be amplified when stronger (e.g. footshock)^31–33^ rather than weaker (e.g. omitted food, non-painful air-puff)^29,30^ aversive stimuli are used. A further factor that applies to both manual and automated tasks is that lack of control over trial initiation can result in trials starting when animals are not attending to the task, or not starting when they are attending and motivated, both of which may interfere with task engagement, decreasing the number of trials completed^34^.

**Figure 1:**
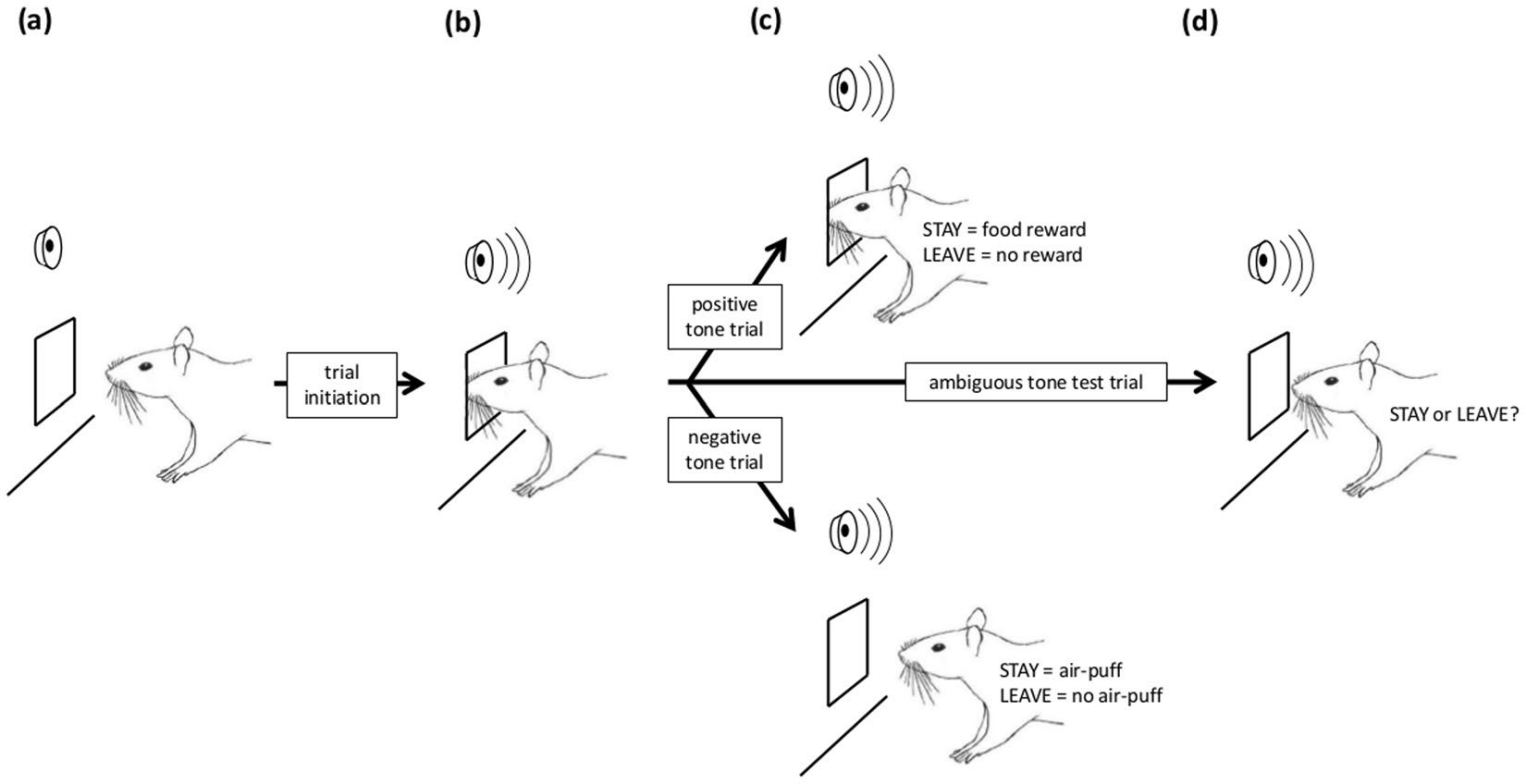
Schematic of the judgement bias task. (**a**) The rat is free to initiate a trial by nose-poking into the food trough recess whenever it wants. (**b**) The rat initiates a trial and a tone sounds. During training sessions (**c**) this can either be the ‘positive tone’ or the ‘negative tone’. If the positive tone sounds, the rat must STAY for 2 s with its head in the food trough to receive a food reward. If it LEAVES before 2 s has elapsed, the food is not dispensed. If the negative tone sounds, the rat must LEAVE the trough within 2 s to avoid an air-puff delivered into the trough recess. If it STAYS for longer than 2 s, the air-puff is presented until the rat withdraws from the trough. Once this discrimination is learnt, testing sessions include presentations of occasional intermediate (ambiguous) tone trials (**d**). On these trials, the STAY response is interpreted as the rat anticipating a positive outcome (food) and the LEAVE response as it anticipating a negative outcome (air-puff). See text for further details.

Given this background, our aim was to employ standard operant equipment to develop an automated judgement bias task that rats could learn relatively quickly. To this end, we capitalised on the rat’s natural ‘nose-poking’ investigative behaviour^35^ to design a task in which response requirements involved poking into and withdrawing from a food trough (Fig. 1). Subjects self-initiated trials by inserting their nose into the food trough whereupon a discriminative cue (a tone) was presented. If the cue predicted a positive outcome, rats had to keep their nose in the trough for 2 s to receive a food pellet. However, if the cue predicted a negative outcome, they had to remove their nose within 2 s to avoid delivery of an air-puff in the trough (we chose this non-painful stimulus in accordance with 3Rs principles, and to minimise any Pavlovian constraints on learning an active response to it). We reasoned that self-initiation should ensure that rats are engaged with the task on each trial, thus making a meaningful response, and that the absence of enforced, and potentially frustrating, inter-trial intervals should permit a large number of trials to be completed per day. Likewise, we expected that the use of natural investigative behaviour should increase task engagement and learning speed. It should also be easily translatable across taxa because approach towards, and investigation of, a potential source of reward, coupled to withdrawal from an aversive stimulus, are likely to reflect Pavlovian predispositions in many species^30^. We also investigated ways of addressing one of the task’s potential difficulties; that subjects may learn the outcome of ambiguous cue trials and alter their responses accordingly (e.g. extinction of responses to non-reinforced cues^36^). To do this, we compared the effectiveness of partially-reinforced discrimination training combined with non-reinforced ambiguous cues, to that of fully-reinforced training combined with randomly reinforced ambiguous cues.

As a first step in demonstrating construct validity of the task, we employed short-term affective manipulations that both reflect husbandry and procedures experienced by experimental animals (Experiment 1), and also theoretical predictions concerning the links between frequency of recent experience of positive and negative events and judgement bias (Experiment 2). In Experiment 1, we exposed rats to treatments assumed to induce relatively positive (gentle handling, enrichment) or negative affective states (restraint, isolation). Rats will work to gain access to a familiar handler^37^, work to gain access to enriched cages containing a nest box compared to empty cages^38^, and display positive judgement biases when housed in enriched as opposed to barren environments^20,22,39^. Rats placed in an unfamiliar cage^40^ or a restraint tube or cone^41^ show physiological stress responses including elevated body temperature, corticosterone, heart rate and blood pressure with the latter two being resistant to habituation across repeated cone-restraint trials^42^.

In Experiment 2, the affect manipulation was predicated on the Bayesian reasoning mentioned above according to which repeated positive (respectively negative) experiences would be predicted to generate a positive (negative) affective state and associated increased expectation of positive (negative) outcomes in the future. Accordingly, two manipulations were used – high and low reward frequency during the period preceding the judgement bias test.

## Materials and Methods

### Experiment 1

#### Animals

The subjects were 40 male Lister Hooded rats (*Rattus norvegicus*, Harlan Ltd, UK), aged 10 weeks on arrival and housed in pairs in 1500U Eurostandard type IV S cages (480(L) × 375(W) × 210(H) mm) in ventilated Scantainer cabinets (Scanbur, Denmark), under a 12 h reversed light-dark cycle (lights on 1900-0700). See Supplementary Information for power analysis details. Cages contained sawdust substrate, shredded paper bedding and two cardboard tubes. Food (LabDiet) and water were available *ad libitum*. All rats were checked regularly for any health issues, and the work was approved and conducted under UK Home Office licence 30/2954.

#### Apparatus

We used four identical shuttle boxes (508 × 254 × 305 mm) each placed in a sound isolation chamber. Each box was divided in half by a metal panel and one half (254 × 254 × 305 mm) was used for the judgement bias test with the other half closed off. A loudspeaker was positioned centrally on top of the operational half of the shuttle-box, and a food trough supplied by an automated food dispenser delivering Bioserv (USA) Dustless Precision Pellets (45 mg sucrose) was located on the end wall and was accessible through a 32(W) × 40(H) mm opening 35 mm above the floor. Air-puffs could be delivered from a tube located on the right-hand side of the trough recess and connected, via Tygon tubing and a control unit containing a 50 psi solenoid, to a compressed air cylinder (BOC UK). All hardware was manufactured by Coulbourn Instruments (Allentown, PA, USA), and operated by their Graphic State (v4) software.

#### Judgement Bias Training

Training and testing were carried out in the dark phase of the light cycle, and rats were not food-restricted during training or testing. Following a habituation procedure (see Supplementary Information), rats received one training session per day, five days per week. The aim of the first phase – *positive tone training* – was to train rats that when they heard the positive tone (2 kHz at 76 dB for one rat in each cage pair and 8 kHz at 65 dB for the other (cf.^32^)), they should keep their nose in the trough in order to receive a sucrose pellet reward (a ‘stay’ response). Rats initiated trials by placing their nose into the food trough recess thus breaking a photo-sensor beam. On 50% of trials, the positive tone sounded and if the rat kept its nose in the recess for a pre-determined duration (increasing from 20 ms to 0.5 s, 1 s, 1.5 s across sessions), during which the tone continued to sound, a sucrose pellet was delivered and the tone stopped. If they withdrew before the duration was complete, the tone stopped and they did not receive a pellet. On the other 50% of trials, no tone was played (null trial) and no food was delivered. The aim was to train an association between the positive tone and reward. Each session lasted 15 min, and two trials of each type were presented randomly in each block of four trials. Rats had to keep their noses out of the trough recess for at least 1 s before the next trial started. From session 3 onwards, rats that initiated at least 15 positive tone trials in a session had the required nose poke duration extended to 0.5 s for the following session (e.g. session 4). If they then initiated at least 15 positive tone trials and made a ‘stay’ response on at least 62.5% of these on each of two consecutive sessions, the nose poke duration was increased to 1 s. For the duration to be increased to 1.5 s, the same criterion needed to be achieved, and for it to then be increased to 2 s, the same criterion but with a 70% ‘stay’ response rate was required. Thus, a minimum of 9 sessions was required to get to the 2 s nose poke duration, and at this point rats moved to the next phase of training.

In the second phase – *discrimination training* – rats initially experienced sessions comprising 40% positive trials, 40% null trials, and 20% negative trials. When they heard the positive tone they had to keep their head in the food trough recess for 2 s, during which the tone continued, in order to obtain a sucrose pellet. When they heard the negative tone (8 or 2 kHz – opposite to their positive tone) they needed to withdraw (a ‘leave’ response) in order to stop the tone sounding and avoid a 50 psi air-puff into the trough recess. If the rat failed to withdraw within 2 s they received the air-puff which stopped 1 s after they removed their snout from the trough. The first two discrimination training sessions were 15 min long and this was increased to 30 min from the third session onwards. Once rats initiated at least 10 positive trials per session and achieved at least 62.5% correct, ‘stay’ and ‘leave’ respectively on both positive and negative trials, over two consecutive sessions, sessions were altered to comprise equal quantities (33% each) of positive, negative and null trials. When rats achieved the same criterion on these sessions, they moved to the final training phase. This could thus be achieved in a minimum of 4 sessions.

In the final phase – *fully- or partially-reinforced discrimination training* – one rat in each cage was allocated to fully-reinforced (FR) training and the other to partially-reinforced (PR) training, counter-balanced with positive tone frequency (2 vs 8 kHz) across cages. For both sets of rats, 45% of trials were positive, 45% negative, and 10% null, and, for FR-trained rats, these were reinforced as previously. For PR-trained rats, the same ratio of trial types was used but 22% of positive and 22% of negative trials were not reinforced; a correct ‘stay’ response to a positive tone would not be rewarded with food, and an incorrect ‘stay’ response to a negative tone would not result in air-puff delivery. Therefore, in total, 35% of trials were reinforced positive, 35% were reinforced negative, 10% were null, 10% were unreinforced negative, and 10% were unreinforced positive, with trial order randomised. When rats achieved 70% correct on both positive and negative trials over two consecutive sessions, and initiated at least 30 trials of each type per session, they moved on to the judgement bias testing phase of the study. This could be achieved in a minimum of 2 sessions.

#### Judgement Bias Testing

In judgement bias tests, we used a single ambiguous tone (4 kHz at 71 dB) which was assumed to be close to the perceived mid-point between the training tones given that the perception of sound frequencies follows an approximately logarithmic distribution^43^. Each test session comprised 6 blocks of 11 trials. Within each block, rats trained on full-reinforcement (FR) received, in random order, 4 reinforced positive and 4 reinforced negative trials, 1 additional reinforced positive or negative trial, 1 null trial, and 1 reinforced ambiguous trial such that, across all 66 trials, they experienced 27 of each of reinforced positive and reinforced negative trials, 6 null trials, and 3 ambiguous trials that were positively reinforced and 3 that were negatively reinforced (reinforcement only occurred when rats made ‘stay’ responses). For rats trained on partial-reinforcement (PR), each block of 11 trials was structured in the same way except that the additional positive or negative trial and the ambiguous trial were not reinforced. Thus, across all 66 trials, they received 24 of each of reinforced positive and reinforced negative trials, 3 of each of unreinforced positive and negative trials, 6 null trials, and 6 unreinforced ambiguous trials. Test sessions finished after 40 min or when 66 trials were completed.

#### Affect Manipulations and Judgement Bias Tests

Once rats had completed the final phase of judgement bias training, they began 7 weeks of affect manipulations and judgement bias testing. Each week, each rat took part in one discrimination training session exactly the same as those that they had experienced in the final phase of training and, two days later, one judgement bias testing session. In weeks 2, 3, 5 and 6 rats were exposed to four different affect manipulations, counter-balanced for order across subjects, in the 15 min before they were exposed to the judgement bias test.

Two of the manipulations were designed to induce a positively-valenced affective state. These were (i) *gentle handling* – exposure to 15 min gentle handling in which a familiar person gently picked up the rat, allowed it to climb freely over them while seated, and calmly stroked the animal during the last 9 min; (ii) *enrichment* – 15 min exposure to an enriched area containing sand, straw, tunnels, rope and a warm water bath which the rats could choose to use or not. The other two manipulations were designed to induce a negative affective state: (i) *restraint* – the rat was placed in a plastic restraining box (195 × 105 × 130 mm) within a larger holding cage in an empty room for 15 min; (ii) *isolation –* the rat was placed on its own in an unfamiliar standard cage (same as home cage but with sawdust substrate only) in an empty room for 15 min.

### Experiment 2

Methods were largely the same as for Experiment 1. Differences are summarised below and mainly reflect changes in the light of findings from the first experiment.

#### Animals

Subjects were 12 male Lister Hooded rats, aged c.5mo when training commenced. They had taken part in two behavioural studies prior to this experiment, neither of which involved testing in operant chambers, and were housed in stable groups of three in cages measuring 560(L) × 340(W) × 190(H) mm within a ventilated Scantainer cabinet under a 12-hour reversed light-dark cycle (lights on 0815–2015). Cages contained one chew block, three cardboard tubes, and one plastic tube attached to the cage ceiling. Food and water were available *ad libitum*. All rats were checked regularly for any health issues, and the work was conducted under University of Bristol Investigation Number UB/16/004 following approval by the Bristol University Animal Welfare and Ethics Review Body.

#### Apparatus

Operant chambers and software were as described for Experiment 1. A clear Perspex box measuring 400 × 250 × 300 mm with a clear plastic food pot of 30 mm diameter placed centrally was used for the reward experience manipulation.

#### Judgement Bias Training

The training protocol was as for Experiment 1 except for a few minor adjustments. During *positive tone training*, 2 kHz was the positive tone for all rats due to an observed effect of tone contingency adding noise to the data from Experiment 1 (see Results). The required time for rats to keep their nose in the trough recess (‘stay’) in order to receive a food pellet progressed from 20 ms to 0.75 s to 1.5 s across sessions and so rats could complete this phase in a minimum of 7 sessions. During *discrimination training*, the required ‘stay’ time was increased to 2 s. The first three sessions were 15 min long with an increase to 30 min from the fourth session onwards. Once rats achieved criterion performance on the 30 min sessions with 40% positive trials, 40% null trials, and 20% negative trials, they went through one session in which this was altered to 45% positive, 45% negative, 10% null, before moving to *partially reinforced (PR) discrimination training*. A minimum of 6 sessions was thus required to complete this training phase. PR training was selected for all rats due to the influence of FR training observed in Experiment 1 (see Results). When rats achieved criterion for 2 consecutive sessions, they moved to the judgement bias test.

#### Judgement Bias Testing

For judgement bias tests, we used three ambiguous tones (2.8 kHz (74 dB), 4 kHz (71 dB), 5.6 kHz (68 dB)) rather than one because the single tone used in Experiment 1 did not appear to be perceived as the mid-point between the two training tones (see Results). Ambiguous tone trials were not reinforced and made up 30% of trials (18/60; 6 trials of each tone). 35% (21/60) of trials were reinforced positive, and 35% were reinforced negative. Test sessions finished after 40 minutes or when 60 trials had been completed.

#### Affect Manipulation and Judgement Bias Test

The affect manipulation involved exposing rats to a high reward frequency (H) or low reward frequency (L) immediately before the judgement bias test. Rats experienced the reward frequency manipulation in the reward experience box described earlier. All rats had been given 20 min prior experience of the box at the start of the discrimination training phase and were placed in it for an initial 5 min to reacclimatise before the start of the manipulation. 50% of rats were randomly allocated to the H group and received 16 sucrose pellets delivered on the minute (starting at 0 min) over 15 min. The rats in the L group received just one pellet, with sham pellet deliveries occurring every minute starting at 0 min, with the exception of the 8^th^ minute at which the sucrose pellet was delivered. Immediately following the reward experience treatment, rats undertook the judgement bias test. Rats were tested once.

### Statistical Analysis

For judgement bias training, the effects of reinforcement schedule (FR vs PR) and tone frequency associated with the sucrose pellet (8 kHz vs 2 kHz) on the number of sessions to criterion were analysed for Experiment 1 data using Kaplen-Meier survival analysis in IBM SPSS statistics version 24.

For judgement bias testing, the key outcome variable was the *response* (‘stay’ or ‘leave’) made on trials to the presented cue. An additional variable of interest was the subject-controlled inter-trial-interval (*ITI*) between the end of one trial and self-initiation of the next which may indicate the rat’s vigour or motivation for reward during testing. Generalised linear mixed models (GLMMs), which included a random effect of session nested within individual nested within cage, were fitted to the *response* and log-transformed *ITI* data using the nlme and lme4 packages in R^44,45^ (see^46^). The model of the response data used a binomial error structure, and the model of the log-transformed ITI data assumed a gaussian error structure. Following verification of the model assumptions, likelihood ratio tests (LRT) were used to assess whether the difference in model deviance was significant when a predictor variable was removed from the model. Each n-order term was dropped, non-sequentially, from the full n-order model in turn and then compared to the full n-order model using a LRT.

The aim of our analysis was to explore how judgement bias was affected by key methodological considerations and the affect manipulations used. To this end, we fitted a binomial GLMM to the *response* (‘stay’ vs ‘leave’) data on each trial of each judgement bias test session with the following predictor variables for Experiment 1: reinforcement schedule (FR vs PR), tone frequency associated with the sucrose pellet (8 kHz vs 2 kHz), treatment valence (positive vs negative), treatment type (handling (gentling/restraint) vs environment (enrichment/isolation)), cue presented on that trial (positive vs ambiguous vs negative). We analysed interactions between the first four variables and cue presented to search for changes which were specific to the ambiguous cue, and we investigated whether the combination of treatment type and valence influenced responses by analysing their interaction, and the treatment valence, type, cue interaction. The number of sessions completed and trial number within a session were included in the model as control variables to account for inter-session variability (e.g. change in performance across repeated test sessions) and inter-trial variability (e.g. change in performance within a test session). The 6 null trials per session in which no tone was played were not analysed and sessions therefore included 60 trials in total. Sessions were excluded from the analysis if a rat completed fewer than 5 out of 6 ambiguous trials (83.33%). Rats did this in only 4 out of 124 test sessions (2 ambiguous trials: rat 19; 3 trials: rats 27 and 33; 4 trials: rat 33), completed 5 ambiguous trials in a further 4 sessions (3 for rat 5 and 1 for rat 33), and completed all 6 ambiguous trials in the remaining 116 sessions.

We fitted a gaussian GLMM to the log-transformed inter-trial interval (ITI) data to explore the influence of affect manipulations and methodological factors on how quickly a rat initiated a trial following the previous trial. The same predictors (the cue was that presented on the trial *preceding* the ITI), interaction terms, random effects, and dataset were used as in the analysis of the responses made on each trial, controlling for the number of sessions completed and trial number within a session.

To explore whether pre-test reward experience influenced judgement bias in Experiment 2, we fitted a binomial GLMM to the response data with cue presented (2, 2.8, 4, 5.6, 8 kHz), pre-test reward frequency (high vs low), and the interaction between these variables as predictor variables. The model additionally controlled for trial number within the session. A gaussian GLMM was fitted to the log transformed ITI data to evaluate the effects of the same predictors and interactions on the self-determined inter-trial intervals. As for Experiment 1, sessions were excluded from the analysis if a rat completed fewer than 83.33% (15 out of 18) of ambiguous trials. This was the case for only 1 rat.

Post-hoc pairwise comparisons were conducted using Tukey’s honest significant difference (HSD) test. Where interaction terms which included the variable ‘cue presented’ were found to be significant, the data were split by cue and analysed using further GLMMs. The p-values obtained from these additional analyses were adjusted using the false discovery rate (FDR) method^47^.

## Results

### Experiment 1

#### Judgement Bias Training

**Figure 2:**
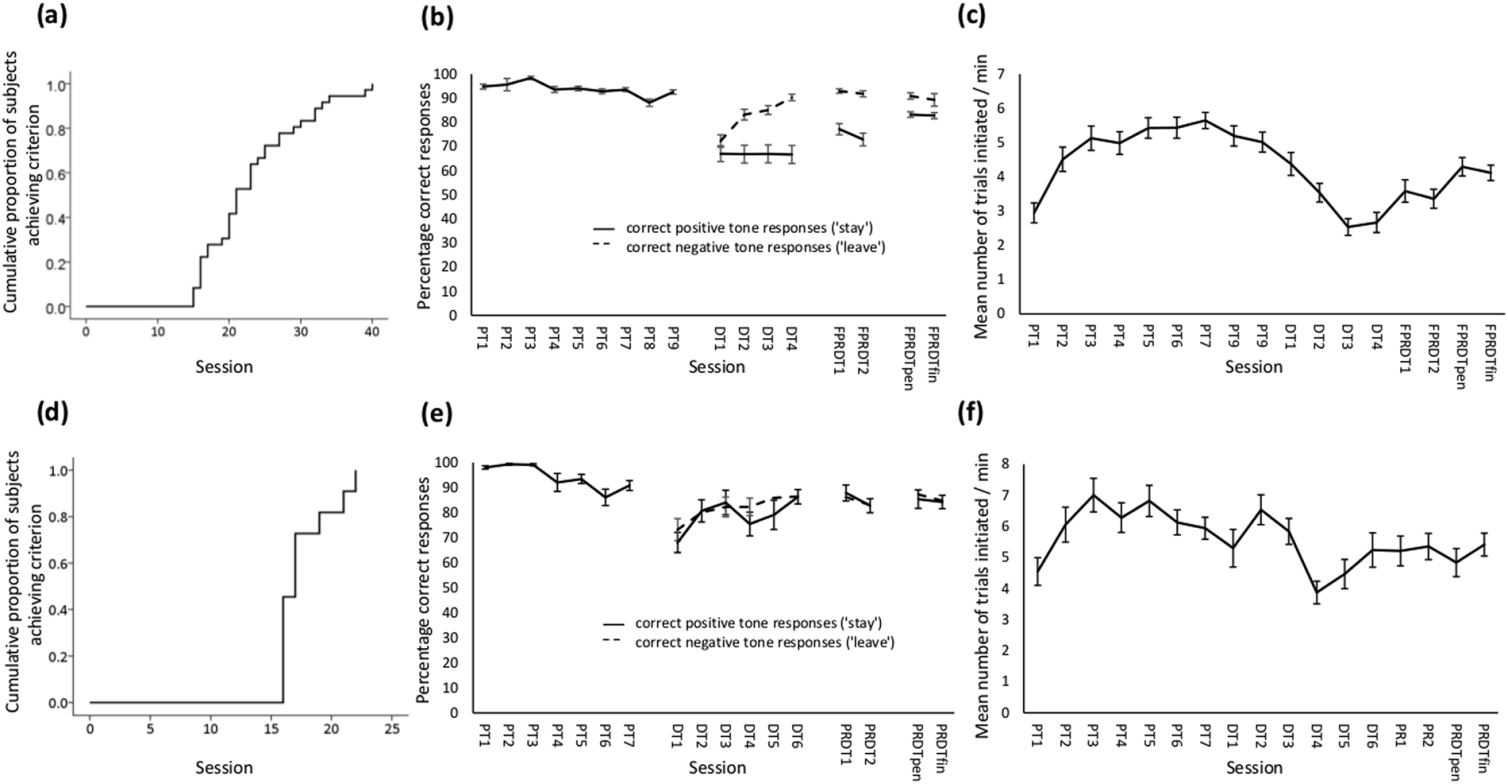
Kaplan-Meier survival curves showing the cumulative proportion of subjects achieving criterion for rats who completed all training phases in (**a**) Experiment 1 and (**d**) Experiment 2. Percentage correct trials during positive training (PT), discrimination training (DT), and full/partial reinforcement discrimination training ((**F**)PRDT) sessions for (**b**) Experiment 1 and (**e**) Experiment 2. Means+/− sem are shown for those sessions before criterion could be achieved during which all rats were participating, and for the penultimate (pen) and final (fin) training sessions before judgement bias testing. Correct responses to positive tones (‘stay’) are shown by solid lines and to negative tones (‘leave’) by dashed lines. Mean number of trials (+/− sem) initiated per minute in training sessions before judgement bias testing for (**c**) Experiment 1 (PT1-DT2 are 15 min long; DT3-FPRDTfin are 30 min long) and (**f**) Experiment 2 (PT1-DT3 are 15 min long; DT4-PRDTfin are 30 min long).

The number of sessions that each rat took to reach criterion is shown in Table S1 in the Supplementary Information for each training phase. During the *positive tone training phase*, all 40 rats had three sessions with 20 ms delays and most then had two sessions at each of 0.5, 1 and 1.5 s delays (i.e. achieved criterion as quickly as possible (9 sessions)). Four animals (2, 11, 14, 18) required extra sessions (4, 2, 1, 1 respectively) to achieve criterion, and only one animal (2) did not proceed on to the next phase due to a low number of trials initiated. Excluding this animal, the mean number of sessions to criterion for this phase was 9.1 (standard error of mean: 0.06). Thirty-six of 39 rats completed the *discrimination training (DT) phase* in a mean of 8 (0.68) sessions, and the *fully- or partially-reinforced discrimination training (FPRDT) phase* in a mean of 5.97 (0.87) sessions. The total number of training sessions before progressing to judgement bias testing is shown for these 36 rats in Fig. 2a (mean: 23.08 (1.14) sessions). There was no significant effect of positive tone frequency (2 vs 8 kHz) on the number of sessions to criterion during the DT phase (Log-rank chi-square = 0.10, df = 1, p = 0.919), the FPRDT phase (Log-rank chi-square = 0.765, df = 1, p = 0.382), or across both phases (Log-rank chi-square = 0.239, df = 1, p = 0.625). Likewise, the number of sessions to criterion during the FPRDT phase (Log-rank chi-square = 0.683, df = 1, p = 0.409) and across both phases (Log-rank chi-square = 0.082, df = 1, p = 0.775) were not affected by reinforcement schedule (FR vs PR). Equipment malfunction (principally air-puff staying on after rats had withdrawn from the trough) slowed the progress of some rats (3, 7, 13, 16, 27). Mean percentage correct responses to positive (‘stay’) and negative (‘leave’) tones and mean number of trials initiated per minute are shown in Fig. 2b,c for those sessions before criterion could be achieved in which all rats participated, and for the final two training sessions before judgement bias testing.

### Affect Manipulation and Judgement Bias Testing

**Figure 3:**
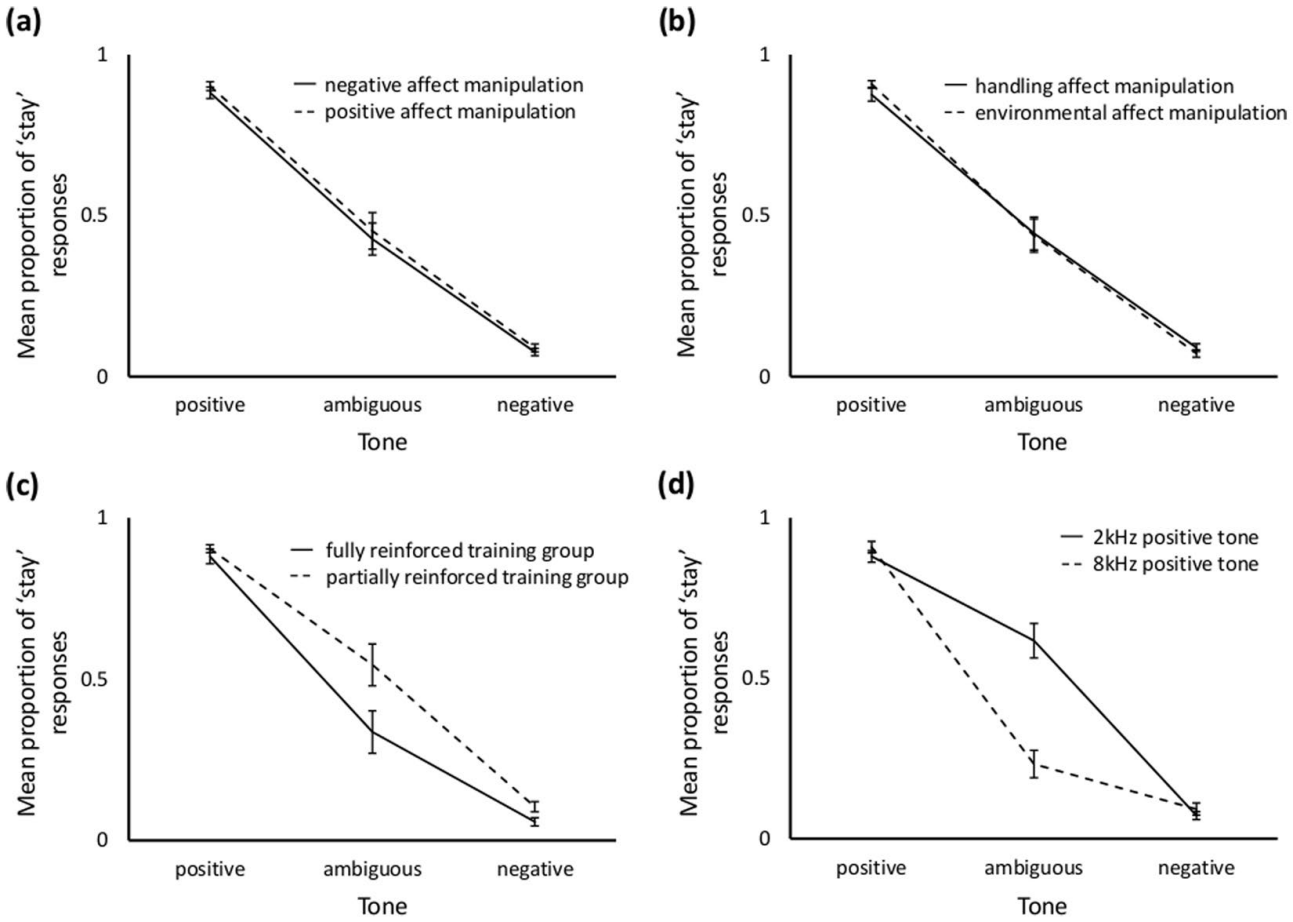
Mean+/− sem proportion of ‘stay’ responses made by rats to training and ambiguous tones during Experiment 1 judgement bias tests according to (**a**) valence (negative = solid line; positive = dashed line) and (**b**) type of affect manipulation (handling = solid line; environment = dashed line), and whether the rats were in the (**c**) fully (solid line) or partially (dashed line) reinforced training groups or (**d**) had 2 kHz (solid line) or 8 kHz (dashed line) as the positive tone.

Thirty-one of 36 rats completed all judgement bias tests. Five rats (20, 32, 35, 36, 38) failed to do so for a variety of reasons (stopped performing for no obvious reason (20) or due to equipment malfunction (32); culled due to illness (35, 36); decreased performance on the positive tone to below 70% correct (38)). Rats experiencing the positively-valenced treatments (gentle handling, enrichment) tended to be more likely to make the ‘stay’ response (LRT = 3.28, p = 0.070; Fig. 3a), but there was no significant interaction between treatment valence and cue presented (LRT = 0.118, p = 0.943). As expected, cue presented had a highly significant effect on response (LRT = 4958.2, p < 0.001); ‘stay’ responses being highest for the positive tone, lowest for the negative tone and intermediate for the ambiguous tone (Fig. 3). There was no main effect of treatment type (LRT = 0.402, p = 0.526), but there was a significant interaction between treatment type and cue presented (LRT = 8.31, p = 0.016; Fig. 3b), although post-hoc analysis failed to detect differences between treatments at any tone (positive: LRT = 0.264, p = 0.156; ambiguous: LRT = 0.001, p = 0.973; negative: LRT = 3.93, p = 0.14 (FDR-adjusted p-values)). No significant interaction was found between treatment valence and treatment type (LRT = 0.118, p = 0.731), or treatment type, treatment valence, and cue presented (LRT = 2.69, p = 0.26).

Rats who experienced fully reinforced (FR) training and ambiguous cue reinforcement were less likely to make the ‘stay’ response than those who experienced partial reinforcement (PR) training and non-reinforced ambiguous cues (LRT = 10.11, p = 0.001). The interaction between reinforcement schedule and cue presented was also significant (LRT = 17.71, p < 0.001; Fig. 3c), and FDR-adjusted post-hoc tests showed that FR rats were less likely than PR rats to make the ‘stay’ response when the ambiguous (LRT = 10.13, p = 0.004) or negative (LRT = 7.258, p = 0.011) tones were presented, but not when the positive tone was presented (LRT = 0.482, p = 0.488).

The tone frequency associated with the sucrose pellet did not predict response (LRT = 1.277, p = 0.258), but there was a significant interaction between this variable and cue presented (LRT = 123.1, p < 0.001; Fig. 3d). FDR-adjusted post-hoc tests indicated that rats trained to associate the 8 kHz tone with reward were less likely to stay in the trough when the ambiguous tone was presented (LRT = 26.01, p = 0.0000012) than rats trained to associate the 2 kHz tone with reward, but not when the positive (LRT = 1.834, p = 0.264) or negative (LRT = 0.255, p = 0.614) tones were presented. The analysis controlled for any effect of session and trial number. Session itself had no significant effect on responses (LRT = 0.487, p = 0.485), including when ambiguous cues were analysed on their own (LRT = 0.0042, p = 0.949), but trial number did (LRT = 5.04, p = 0.025 (although not for the ambiguous cue alone; LRT = 1.337, p = 0247)) with a beta-coefficient of −0.005 and no clear cross-time change discernible from data plots (Supplementary Information, Fig. S1).

**Figure 4:**
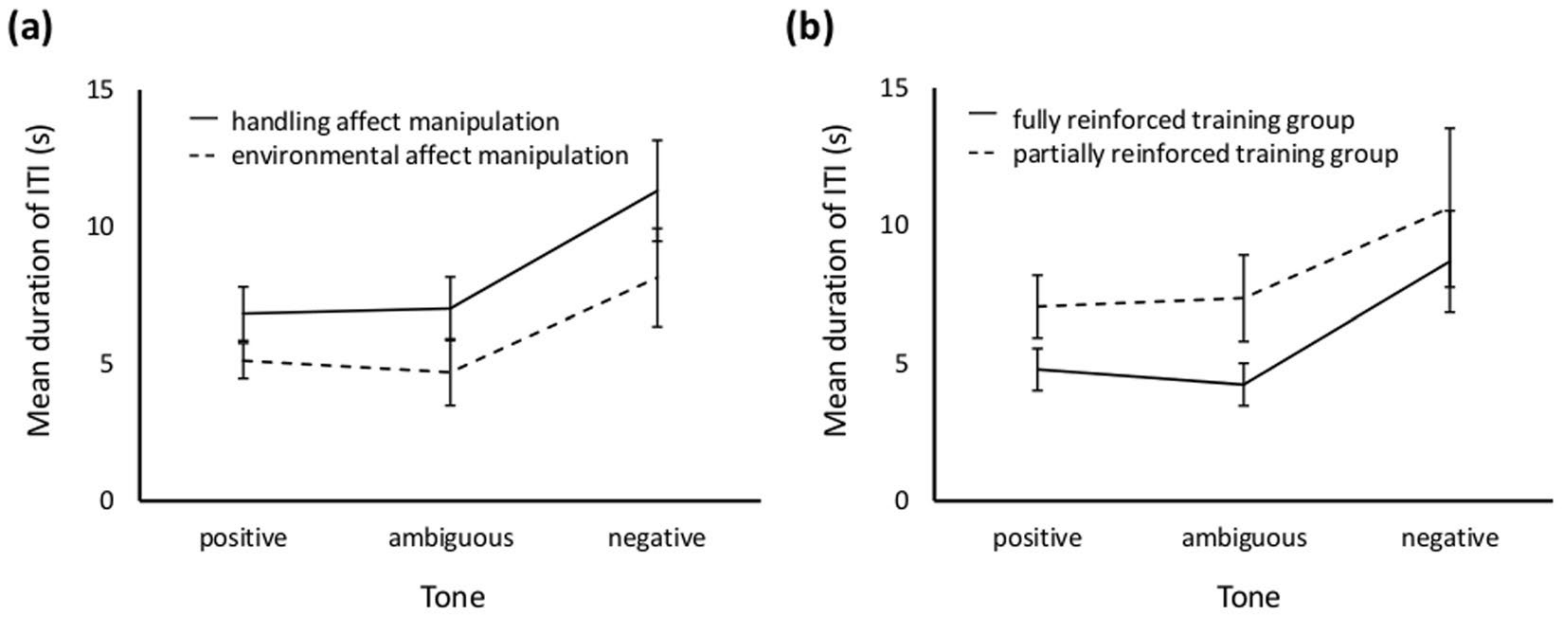
Mean+/− sem duration of self-determined inter-trial intervals across training and ambiguous tones during Experiment 1 judgement bias tests in rats exposed to (**a**) handling (solid line) or environmental (dashed line) affect manipulations, and (**b**) in rats in the fully (solid line) or partially (dashed line) reinforced training groups.

Analysis of the inter-trial interval (ITI) data showed that rats had significantly shorter ITIs following the environmental as opposed to handling treatments (LRT = 8.87, p = 0.002; Fig. 4a), but ITIs were not affected by treatment valence (LRT = 0.557, p = 0.455), frequency of the tone associated with the sucrose pellet (LRT = 0.044, p = 0.834), or reinforcement schedule (LRT = 2.47, p = 0.116). The tone presented on the preceding trial had a strong effect on ITI, with ITIs being longer after a negative tone trial (LRT = 37.26, p < 0.001; Fig. 4). The only significant interaction effect was between tone and reinforcement schedule (LRT = 12.07, p = 0.0024; Fig. 4b). FDR-adjusted post-hoc tests indicated that, compared to rats trained with partial reinforcement, those trained with full reinforcement tended to show shorter ITIs following a positive tone trial (LRT = 4.731, p = 0.089), but not after either an ambiguous (LRT = 0.122, p = 0.727) or negative tone trial (LRT = 0.308, p = 0.727). The analysis controlled for any effect of session and trial number. Trial number affected mean ITI (LRT = 37.26, p < 0.001) with some suggestion of an increase in ITI as the session progressed, and there was a tendency for mean ITI to decrease in a rat’s last session (LRT = 2.89, p = 0.089; Supplementary Information, Figs S2, S3).

### Experiment 2

#### Judgement Bias Training

The number of sessions that each rat took to reach criterion is shown in Table S2 in the Supplementary Material for each training phase. Eleven rats completed *positive tone training* (one rat failed to perform enough trials per session). Of these, 10 completed this phase as quickly as possible (7 sessions) with one rat taking 9 sessions (mean: 7.18 (0.18)). One rat completed *discrimination training* in the minimum 6 sessions, 6 rats took 7 sessions, and 4 rats took each of 8–11 sessions (mean: 7.82 (0.46)). Eight rats required the minimum of 2 sessions to complete *partial reinforcement discrimination training*, and 3 rats took each of 3–5 sessions (mean: 2.54 (0.31)). The 11 rats that completed training took a mean of 17.54 (0.65) sessions to progress through all phases (Fig. 2d). Mean percentage correct responses to positive and negative tones and mean number of trials initiated per minute are shown in Fig. 2e,f for those sessions before criterion could be achieved in which all rats participated, and for the final two training sessions before judgement bias testing. Performance was more similar on the two tones than in Experiment 1 (Fig. 2b).

### Affect Manipulation and Judgement Bias

**Figure 5:**
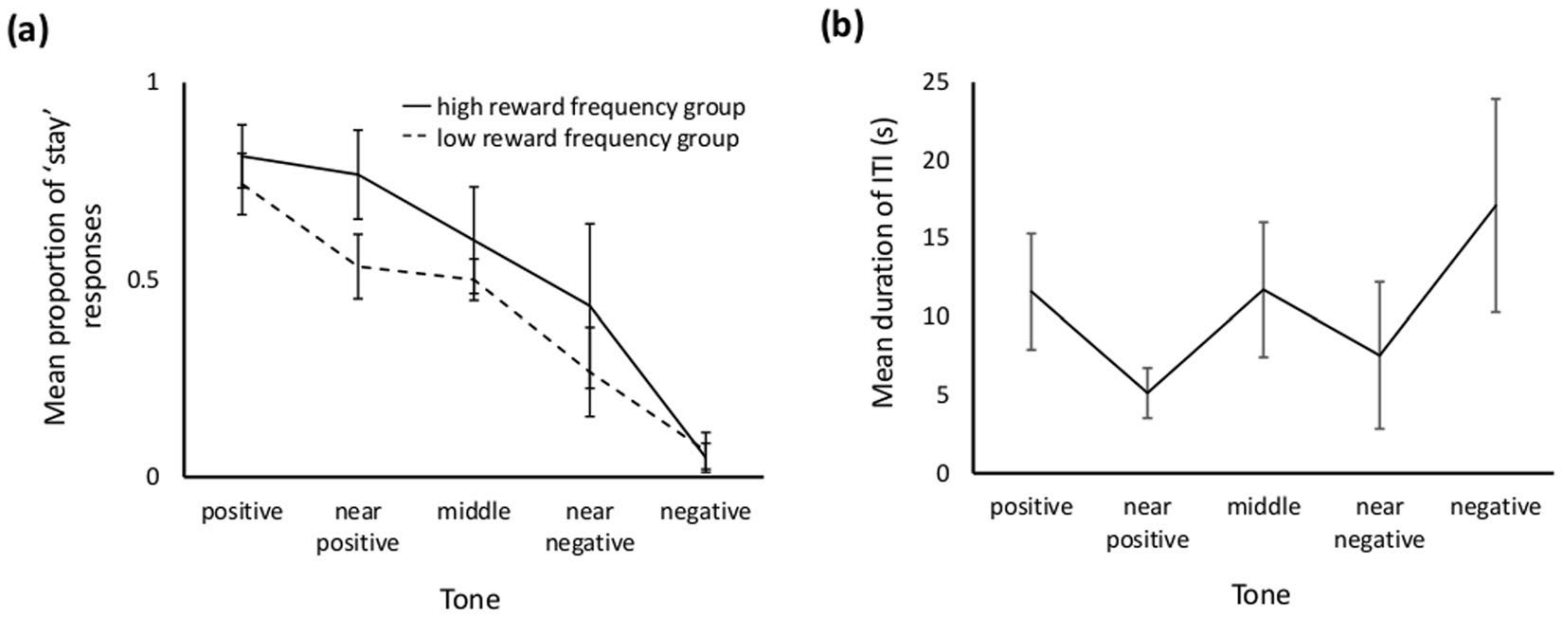
(**a**) Mean+/− sem proportion of ‘stay’ responses made by rats to training and ambiguous tones during Experiment 2 judgement bias tests in the high reward frequency (solid line) and low reward frequency (dashed line) treatment groups. (**b**) Mean+/− sem duration of self-determined inter-trial intervals during Experiment 2 judgement bias tests following trials with tones of different frequencies (positive = 2 kHz; near positive = 2.8 kHz; middle = 4 kHz; near negative = 5.6 kHz; negative = 8 kHz).

Rats that received rewards at a high frequency prior to the test tended to make the ‘stay’ response more than rats that received rewards at a low frequency (LRT = 3.748, p = 0.053; Fig. 5a). As expected, the cue presented had a highly significant effect on response (LRT = 303.33, p < 0.001; Fig. 5a); FDR-adjusted post-hoc tests showed that rats were significantly more likely to ‘stay’ when the tone nearest to the positive tone (2 kHz) in the following pair-wise comparisons was presented (2 vs 4, 2 vs 5.6, 2 vs 8, 2.8 vs 5.6, 2.8 vs 8, 4 vs 8, 5.6 vs 8). There was no interaction between cue presented and pre-test reward frequency (LRT = 3.507, p = 0.477). Trial number within a session was controlled for and had a significant effect on response with rats appearing to become more likely to ‘stay’ as the session progressed (LRT = 15.92, p < 0.001; Fig. S4).

Analysis of the self-determined inter-trial intervals showed that ITIs were not influenced by pre-test reward experience (LRT = 0.103, p = 0.748), but they were affected by cue presented (LRT = 36.27, p < 0.001; Fig. 5b), becoming shorter when the preceding tone had been ambiguous as opposed to a positive or negative training tone for the following pair-wise combinations (2 vs 2.8, 2 vs 5.6, 8 vs 2.8, 8 vs 4, 8 vs 5.6). There was no significant interaction between the two variables (LRT = 1.705, p = 0.79). Trial number within a session was controlled for and rats showed great variation in ITI length across trials with some suggestion of a shortening as sessions progressed (LRT = 5.179, p = 0.023; Fig. S5).

## Discussion

The aims of this study were to develop an automated judgement bias task for laboratory rats that is relatively quick to train, can be implemented using widely available equipment, puts the animal in control to ensure that it is motivated and attending on each trial by using trial-self-initiation involving natural investigative behaviour, and allows for repeated testing. These objectives were largely achieved.

Experiment 1 demonstrated that rats could learn the task in as few as 15 sessions – the minimum required to achieve the researcher-imposed learning criteria. By rewarding the rat’s natural behaviour of investigating objects (in this case the food trough) with its nose, and pairing reward delivery with a tone, performance on the ‘positive’ tone was high from the first session onwards. Eight further sessions were then used to extend the time required for the rat to keep its nose in the trough to 2 s. This could be decreased, as in Experiment 2, to shorten this training phase. When the air-puff and associated ‘negative’ tone was introduced, there was a noticeable decline in performance on the positive tone. At this point, performance was generally better on the negative tone, suggesting that avoiding the air-puff may have been more salient to rats in this study than acquiring a food pellet. This continued to be the case, although was less pronounced, during the final two training sessions before judgement bias testing commenced. Thirty-six of 40 rats completed training (90%) which is comparable to other automated tasks for which completion rates are reported (c.70–100%^31,33,48,49^). At the end of training, the rats’ mean trial initiation rate was c.4/min allowing data from c.120 trials to be collected during a 30 min session. This is without any need for food restriction during training which itself may induce a potentially negative affective state.

Although rats learnt the task in as few as 15 sessions, the mean number of sessions required was 23 and there was wide inter-individual variation. Equipment problems (e.g. air-puff staying on in some sessions) hampering performance in some individuals may partly explain this variation. The speed at which rats completed training was not affected by whether they were exposed to 2 kHz or 8 kHz as the positive tone, or to fully or partially reinforced training trials. Despite variation in performance, rats required a smaller mean number of sessions to complete training than for other automated judgement bias tasks (24–60^29–33,48–50^).

Rats performed as expected in the judgement bias tests by treating the 4 kHz ambiguous tone as intermediate between the training tones and showing just below 50% ‘stay’ responses to it supporting the suggestion that, in this task, avoiding the air puff may have been marginally more important to the rats than gaining a food pellet. There was a non-significant tendency for short-term affect manipulations assumed to generate a positive state (gentle handling, enrichment) to increase the likelihood of rats performing ‘stay’ responses across all tones, compared to negative manipulations (restraint, isolation). However, this was not specific to the ambiguous tone as predicted. Such findings may arise if negative treatments result in rats decreasing their sensitivity to or valuation of reward^51^ leading to a generalised tendency to make more ‘leave’ responses across all tones as opposed, or in addition, to increasing their perceived probability of threat under ambiguity. Disentangling whether affect manipulations influence one or both of reward/punishment valuation and outcome probability estimation may be achieved using separate tests of these constructs (Neville *et al*. in prep), or computational modelling approaches to dissect these possibilities apart^52^. The significant interaction between treatment type (handling (gentling, restraint) vs environmental (enrichment, isolation)) and cue presented may have reflected a very small increase in the likelihood that rats exposed to environmental manipulations made a ‘stay’ response to the positive tone and a ‘leave’ response to the negative tone (Fig. 3b), but post-hoc tests failed to localise this difference to any one tone.

A clear finding of Experiment 1 was that rats trained with 2 kHz as the positive tone were much more likely to make ‘stay’ responses to the ambiguous tone than those trained with 8 kHz as the positive tone. A potential explanation is that rats perceived the ambiguous 4 kHz tone as being more similar to 2 kHz than 8 kHz and hence tended to make the ‘stay’ response to it if they had been trained that 2 kHz predicts food, or the ‘leave’ response if they had been trained that 2 kHz predicts air-puff. Although 4 kHz should be perceptually intermediate if rats perceive sound frequencies on a logarithmic scale^43^, there may be inaccuracies in our knowledge of rat auditory perception that explain this finding. This highlights that, particularly when olfactory, visual, tactile and auditory cues (as compared to spatial ones) are used, it can be difficult to predict how subjects perceive ambiguous stimuli. Thus, although counter-balancing of cue-outcome contingencies is common in judgement bias tasks, it can lead to considerable noise in the data which may mask treatment effects by generating different patterns of responses to ambiguity in the different contingency groups. Consequently, in Experiment 2 we used just one tone-outcome contingency, as has been done in other studies of rats with no obvious detrimental effect^49,53,54^.

Rats exposed to full reinforcement during the training phase received 50% positive (food) and 50% negative (air-puff) reinforcement when they made a ‘stay’ response to ambiguous tones during judgement bias tests. This was done to investigate whether such pseudo-random reinforcement (cf.^32^) would facilitate repeated testing without animals learning the outcome of ambiguous cues, in comparison to the commonly-used non-reinforcement of ambiguous cues. However, the opposite was found and rats who were randomly-reinforced on ambiguous tone trials were much more likely to make ‘leave’ responses to these tones than those who were not-reinforced. This is probably because occasional experience of air-puffs following ambiguous tones was surprising (once rats had mastered the task they were rarely exposed to air-puffs) and facilitated learning of a negative association with these cues. This experience may also have acted as a reminder of the negative tone outcome and hence resulted in more cautious responses to this cue too.

Consequently, we used only partial reinforcement training and non-reinforced ambiguous cue testing in Experiment 2. It would of course also be possible to use fully reinforced training and non-reinforced testing (FRNR) as in other automated versions of the judgement bias task^33,49–51^, or indeed partially reinforced training and randomly reinforced testing (PRRR), and a full-factorial design could help to confirm the cause of the effects on responses to ambiguity observed here. To our knowledge, PRRR has not been used in judgement bias tasks because it would negate the main purpose of partial reinforcement during training which is to allow testing of ambiguous ‘probe’ tones in extinction^22,36^. FRNR could be tried in future but in this study (Experiment 1) reinforcement schedule during training had no effect on sessions taken to achieve criterion performance, and there would be a risk that rats would learn more quickly that ambiguous cues have no consequences, thus decreasing the number of repeated tests that could be carried out^36^.

The judgement bias task used in this study allowed rats to self-determine their inter-trial-intervals (ITI). This is not common in existing tasks, but is a potentially useful additional source of information, for example about the vigour with which rats perform the task which in turn may be related to affective valence associated with positive and negative prediction errors^55^ and/or reward/punishment valuation^56,57^. However, we did not find any effect of treatment valence on ITI. Instead, environmental treatments (enrichment, isolation) resulted in shorter ITIs than handling treatments (gentling, restraint). A speculative post-hoc explanation is that the former increased general activity levels by allowing rats to be active and explore, and that this spilled over in to the testing session. Further studies may reveal whether the ITI measure can provide useful additional information.

There was no effect of session on the proportion of ‘stay’ responses made by rats, including for the ambiguous cue only, indicating that repeated testing is feasible using this task. ITIs showed a tendency to shorten in the last (fourth) judgement bias test, and appeared to increase slightly with trial number during a session, perhaps reflecting increased satiation as a session progressed. Trial number also had a significant effect on the mean proportion of ‘stay’ responses made but no clear pattern of change could be discerned.

In Experiment 2, 11 out of 12 rats (92%) completed training in a maximum of 22 sessions (mean: 17.5), five doing so within 16 sessions. This faster and less variable performance may be partly attributable to the changes made to task design following Experiment 1, and the facts that no equipment problems occurred and the rats had had experience of previous behavioural (although not operant) testing. The positive training phase was two sessions shorter with no adverse effect on performance suggesting that a further shortening of this phase could be achieved. In the discrimination training phase, rats were only exposed to 40% positive trials, 40% negative trials, and 20% null trials, whereas exposure to 33% of each also occurred in Experiment 1; the higher proportion of tone exemplars during an Experiment 2 session may have facilitated discrimination learning. Training speed on this task (mean: 17.5 sessions) was clearly faster than for other automated judgement bias tasks, and performance on positive and negative tones was more similar than in Experiment 1. Mean trial initiation rate at the end of training was c.5/min allowing data from c.150 trials to be collected in a 30 min session, again without using food restriction to motivate the rats.

Rats were less likely to perform a ‘stay’ response to ambiguous tones than to the positive training tone, more likely to perform a ‘stay’ response to ambiguous tones than to the negative tone, and more likely to make a ‘stay’ responses to the ‘near positive’ than to the ‘near negative’ ambiguous tone, indicating generalisation of responses across ambiguous tones as predicted. Subjects experiencing the positive affect manipulation (high reward frequency prior to testing) also tended to perform more ‘stay’ responses, a similar effect to that found in Experiment 1. Further study is needed to establish whether this is a robust finding and to clarify whether it is specific to ambiguous as opposed to training tones (see Fig. 5a). Rats also had shorter self-determined ITIs after ambiguous tone trials compared to after training tone trials. This may reflect invigoration of responses following lack of reinforcement after ambiguous tones. Trial number affected response and ITI but in both cases there was large inter-trial variation and patterns of change across time were difficult to detect.

In summary, across two experiments we developed a fully automated judgement bias task for rats which 90% of subjects were able to learn, could be trained more rapidly than reported for existing automated tasks, employed self-initiation ensuring engagement on each trial, used a 3Rs appropriate non-painful negative stimulus (air-puff) rather than shock, allowed repeat-testing, and removed the potential for non-blinded and observer-biased collection of decision data. Rats generalised their responses across ambiguous tones as predicted, and initial attempts at construct validation provided some evidence of main effects in the predicted direction, but not localised to ambiguous tones. Further validation is required, for example using longer-term and pharmacological affect manipulations, as is investigation of whether self-determined ITIs could provide new measures of reward valuation and/or affective arousal that would help elucidate exactly how affect manipulations influence decision-making under ambiguity. The simplicity of the task and its reliance on natural investigative behaviour mean that it is likely to be easily translatable across species and readily adapted for home-cage testing. In its automated form for rats, and potentially mice, it offers the opportunity for more widespread uptake of the judgement bias approach in laboratory animal research to both detect existing welfare problems and evaluate the effectiveness of 3Rs refinements.

## Electronic supplementary material

Supplementary Information

## Data Availability

Data for all variables will be made available on request.
