# Author Correction: Assessing animal affect: an automated and self-initiated judgement bias task based on natural investigative behaviour

**DOI:** 10.1038/s41598-018-34498-1

**Published:** 2018-11-01

**Authors:** Samantha Jones, Vikki Neville, Laura Higgs, Elizabeth S. Paul, Peter Dayan, Emma S. J. Robinson, Michael Mendl

**Affiliations:** 1 Centre for Behavioural Biology, Bristol Veterinary School, University of Bristol, Langford House, Langford, BS40 5DU UK; 2 Gatsby Computational Neuroscience Unit, University College London, 25 Howland Street, London, W1T 4JG UK; 3 School of Physiology, Pharmacology and Neuroscience, University of Bristol, Biomedical Sciences Building, University of Bristol, University Walk, Bristol, BS8 1TD UK; 4 Present Address: Department of Psychology, University of Portsmouth, King Henry Building, King Henry 1st Street, Portsmouth, PO1 2DY UK

Correction to: *Scientific Reports* 10.1038/s41598-018-30571-x, published online 17 August 2018

This Article contains typographical errors in the Acknowledgements section.

“(NC3Rs: grant number K/00008 × /1 to MM,ESP,PD,ESJR)”

should read:

“(NC3Rs: grant number NC/K00008X/1 to MM,ESP,PD,ESJR)”

