# Supplementary material for: Assessing animal affect: an automated and self-initiated judgement bias task based on natural investigative behaviour
Source: Sci Rep. 2018 Aug 17;8:12400. doi: 10.1038/s41598-018-30571-x (PMC6098098; doi:10.1038/s41598-018-30571-x)

Assessing animal affect: an automated and self-initiated judgement bias task based on natural investigative behaviour

Samantha Jones, Vikki Neville, Laura Higgs, Elizabeth S. Paul, Peter Dayan, Emma S.J. Robinson & Michael Mendl

Supplementary Information

Power analysis

A power analysis was carried out using estimates from Burman et al. 2009 (Physiol. Behav. 98, 345-350) by statistical adviser, Dr Richard Parker. A 2-level random intercepts model was fitted using the multi-level statistical software package MLwiN, with ambiguous probe cue nested within animal. Treatment was added as a binary predictor, and each animal's mean latency to move in response to training cues was added as a covariate. The resulting estimates were then used to conduct a power analysis using the ESRC-funded software package, MLPowSim which repeatedly simulates datasets based on population estimates provided by the user, and derives power estimates from models it fits to these datasets. The analysis suggests that approximately eight animals in each treatment would achieve a power of 0.9. Because this study developed a new judgement bias method with potential for higher variability of data than in previous studies on which the power analysis was based, and because Experiment 1 was initially designed to incorporate systematic variations in the time delay between short-term affect manipulations and judgement bias testing in an independent subjects design, 40 rats (ten animals in each time treatment) were studied. Time constraints prevented the time delay study being carried out and instead rats moved on from Experiment 1 to a higher priority pharmacological manipulation study (in prep.).

Pre-training habituation procedure

Rats were familiarised with handling and sucrose pellets in their home cage for 2 weeks. They were then individually habituated to the test apparatus for 10min on the first day with the sound isolation chamber door open and 15min on the next day with the door closed. The food trough was not accessible on either habituation day, but was thereafter.

Table S1 – Number of sessions that each rat took to achieve criterion performance in each training phase and across all training phases (total sessions) in Experiment 1

Rat	Positive training phase	Discrimination training phase	Fully (FR) or partially (PR) reinforced discrimination training phase	Total sessions
1	9	9	2	20
2	13	NA	NA	NA
3	9	5	2	16
4	9	7	4	20
5	9	7	4	20
6	9	6	8	23
7	9	15	16	40
8	9	5	2	16
9	9	14	2	25
10	9	NA	NA	NA
11	11	4	6	21
12	9	NA	NA	NA
13	9	6	10	25
14	10	15	7	32
15	9	19	2	30
16	9	6	8	23
17	9	5	2	16
18	10	15	4	29
19	9	6	4	19
20	9	5	19	33
21	9	4	2	15
22	9	8	6	23
23	9	4	8	21
24	9	8	4	21
25	9	6	2	17
26	9	9	2	20
27	9	6	12	27
28	9	6	2	17
29	9	4	2	15
30	9	10	2	21
31	9	4	2	15
32	9	5	2	16
33	9	13	2	24
34	9	NA	NA	NA
35	9	12	6	27
36	9	6	19	34
37	9	5	2	16
38	9	16	14	39
39	9	7	7	23
40	9	6	17	32

NA= rat did not progress to this phase of training. The minimum number of sessions required to achieve criterion in each phase was: 9 (positive training phase), 4 (discrimination training phase), and 2 (FR or PR discrimination training phase), giving a total minimum of 15 sessions to complete judgement bias training.

Table S2 – Number of sessions that each rat took to achieve criterion performance in each training phase and across all training phases (total sessions) in Experiment 2

Rat	Positive training phase	Discrimination training phase	Partially-reinforced discrimination training phase	Total sessions
1	7	7	2	16
2	9	6	2	17
3	7	7	3	17
4	7	9	5	21
5	7	8	2	17
6	7	7	2	16
7	7	7	2	16
8	7	7	2	16
9	7	11	4	22
10	7	10	2	19
11	7	7	2	16

The minimum number of sessions required to achieve criterion in each phase was: 7 (positive training phase), 6 (discrimination training phase), and 2 (FR or PR discrimination training phase), giving a total minimum of 15 sessions to complete judgement bias training.

Figure S1
Mean proportion of 'stay' responses made by rats across trials in judgement bias testing sessions in Experiment 1.

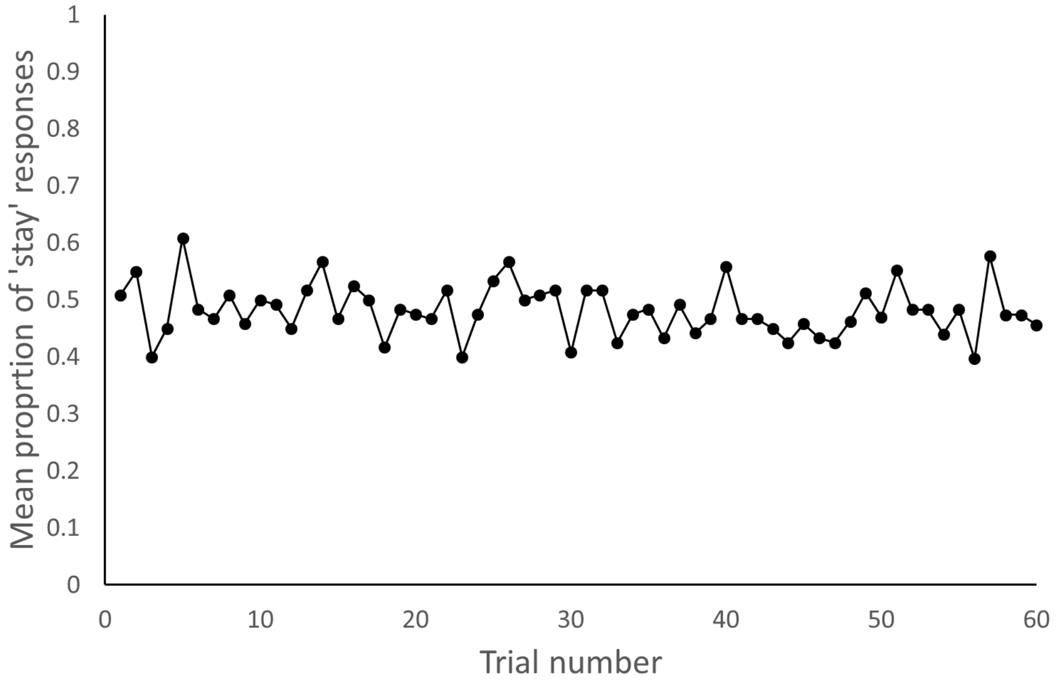

Figure S2
Mean duration of self-determined inter-trial intervals made by rats across trials in judgement bias testing sessions in Experiment 1.

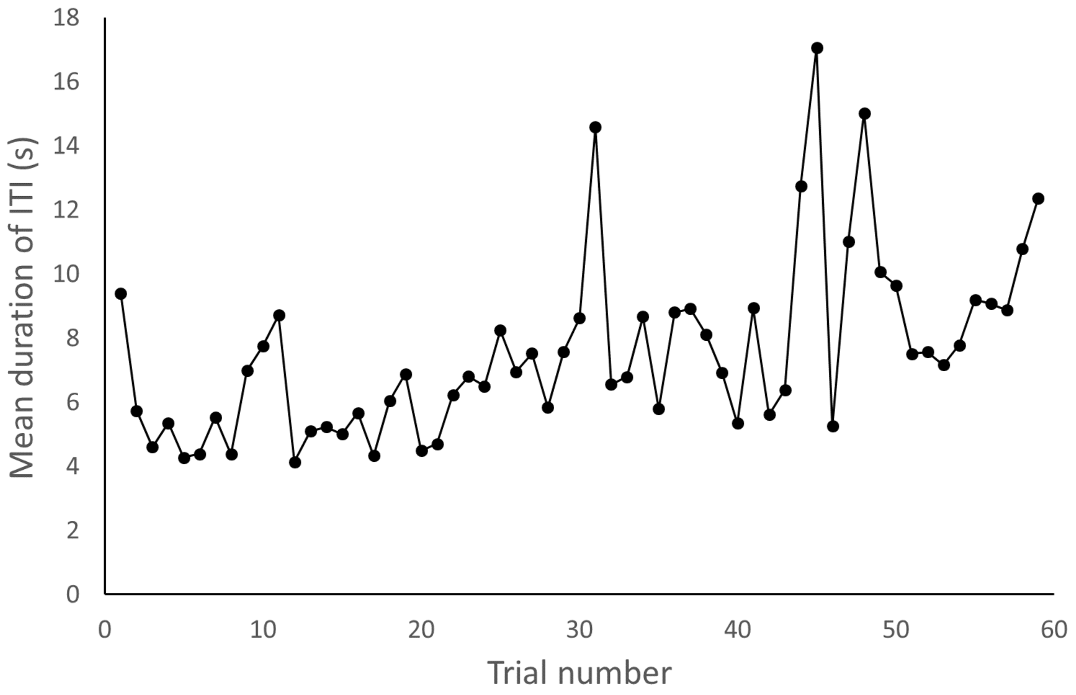

Figure S3

Mean duration of self-determined inter-trial intervals made by rats across judgement bias testing sessions in Experiment 1.

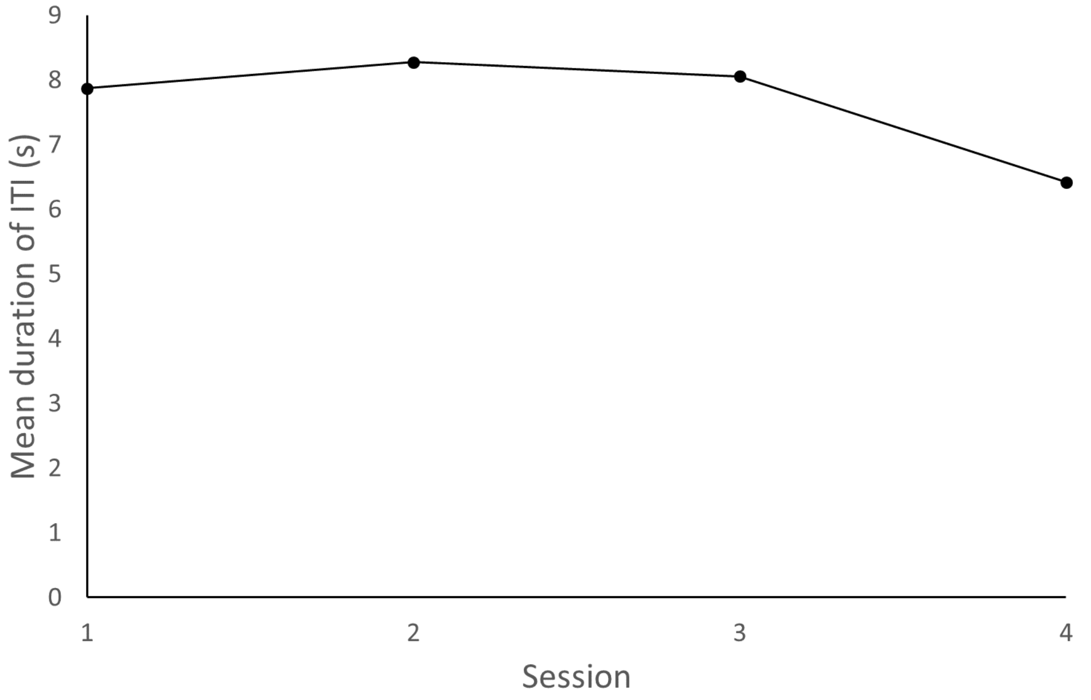

Figure S4

Mean proportion of ‘stay’ responses made by rats across judgement bias testing sessions in Experiment 2.

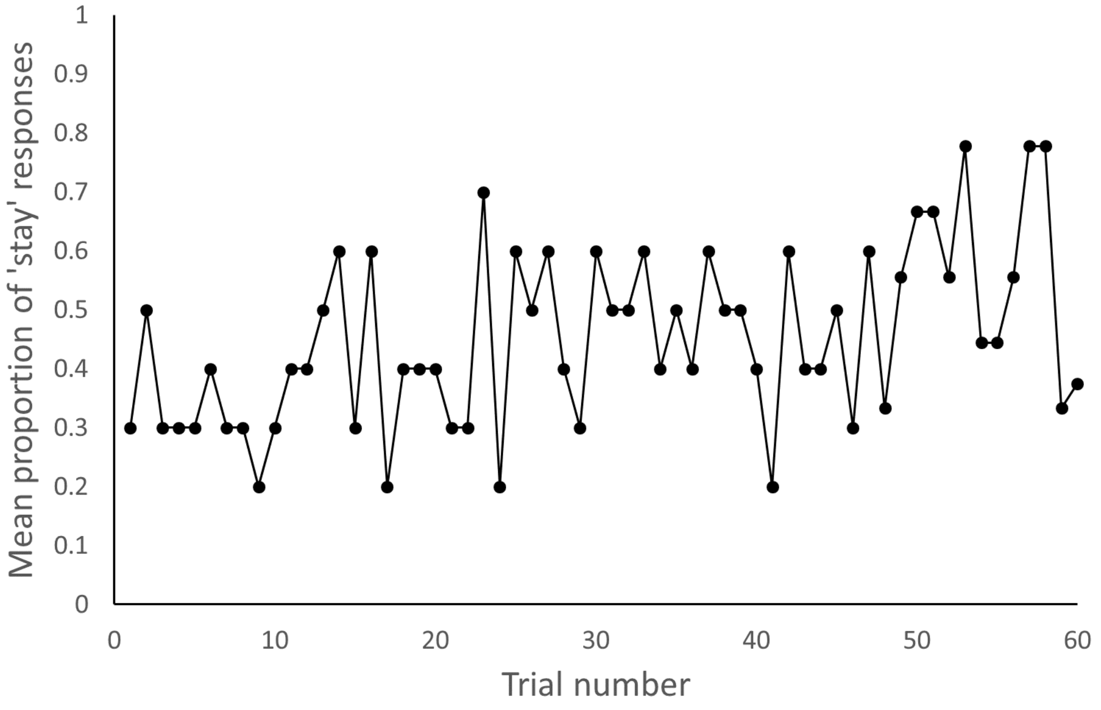

Figure S5
Mean duration of self-determined inter-trial intervals made by rats across trials in judgement bias testing sessions in Experiment 2.

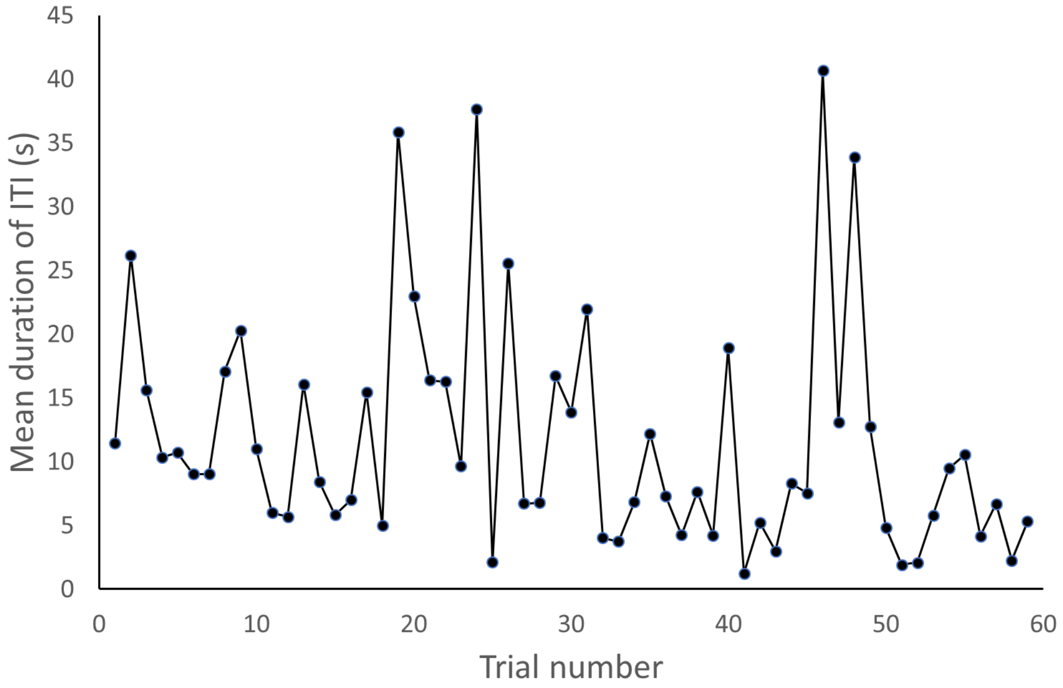

Supplement: Supplementary file 1 — Supplementary Information [file 41598_2018_30571_MOESM1_ESM.pdf]
